# The diagnostic value of cerebrospinal fluid lactate for post-neurosurgical bacterial meningitis: a meta-analysis

**DOI:** 10.1186/s12879-016-1818-2

**Published:** 2016-09-13

**Authors:** Xiong Xiao, Yang Zhang, Liwei Zhang, Peng Kang, Nan Ji

**Affiliations:** Department of Neurosurgery/China National Clinical Research Center for Neurological Diseases, Beijing Tiantan Hospital, Capital Medical University, 6 Tian Tan Xi Li, Beijing, 100050 China

**Keywords:** Cerebrospinal fluid lactate, Post-neurosurgical bacterial meningitis, Diagnostic value, Meta-analysis

## Abstract

**Background:**

Bacterial meningitis is not rare in post-neurosurgical patients. If patients are not treated promptly, the mortality rate can reach 20 to 50 %. The concentration of cerebrospinal fluid (CSF) lactate has been reported to be helpful in the diagnosis of bacterial meningitis; however, no systematic evaluations have investigated CSF from a postoperative perspective. In this study, we performed a systematic evaluation and meta-analysis of the efficacy of using CSF lactate concentrations in the diagnosis of post-neurosurgical bacterial meningitis.

**Method:**

We retrieved studies that investigated the diagnostic value of CSF lactate for the diagnosis of post-neurosurgical bacterial meningitis by searching PubMed, EBSCO, the Cochrane Library and ClinicalTrials.gov. All these databases were searched from inception to November 2015. We used Quality Assessment of Diagnostic Accuracy Studies (QUADAS), a tool for the quality assessment of diagnostic accuracy, to evaluate the quality of the included studies. The Meta-DiSc 1.4 and Review Manager 5.3 software programs were used to analyze the included studies. Forest plots and summary receiver operating characteristics (SROC) curves were also drawn.

**Results:**

Five studies, involving a total of 404 post-neurosurgical patients, were selected from 1,672 articles according to the inclusion criteria. The quality of the five included studies was assessed using QUADAS, and the related results are presented in tables. The meta-analysis revealed the following diagnostic values regarding CSF lactate for post-neurosurgical bacterial meningitis: a pooled sensitivity of 0.92 (95 % CI 0.85–0.96), a pooled specificity of 0.88 (95 % CI 0.84–0.92 with significant heterogeneity), a diagnostic odds ratio of 83.09 (95 % CI 36.83–187.46), an area under the curve (AUC_SROC_) of 0.9601, an SE(AUC) of 0.0122, a Q* of 0.9046 and an SE(Q*) of 0.0179.

**Conclusion:**

The meta-analysis indicated that the CSF lactate concentration has relatively high sensitivity and specificity for the diagnosis of post-neurosurgical bacterial meningitis and thus has relatively good efficacy.

## Background

Bacterial meningitis is not rare in post-neurosurgical patients and has an incidence of approximately 0.3 to 1.5 % [1]. However, the observed incidence in clinical practice is higher than this number. Clinical manifestations such as fever, signs of meningeal irritation and an altered mental status lack specificity and sensitivity [2]. Furthermore, the intraoperative aseptic inflammatory response induced by blood, bone chips, sloughing tissue, and surgical implants as well as the widespread postoperative administration of prophylactic antibiotics increase the difficulty of diagnosing postoperative bacterial meningitis via routine cerebrospinal fluid (CSF) analysis and CSF culture [2–5]. The delayed administration of antibiotics and corticosteroids, as well as the unnecessary administration of these agents, can result in impaired treatment effects [2, 6]. If patients with bacterial meningitis are not treated promptly, the mortality rate can reach 20 to 50 % [6]. Therefore, early and accurate diagnosis is critical for postoperative bacterial meningitis [7].

Previous studies have found that the CSF lactate concentration is associated with bacterial meningitis. The evaluation of CSF lactate levels is relatively efficient in distinguishing between bacterial meningitis and aseptic meningitis [8–11] and is superior to routine CSF analysis [10]. However, post-neurosurgical patients were excluded from Huy’s study [10], and Sakushima did not perform a stratified analysis of the diagnostic value of CSF lactate in postoperative bacterial meningitis [11]. Recent studies have indicated that CSF lactate shows a certain degree of diagnostic accuracy for differentiating between postoperative bacterial meningitis and aseptic meningitis [12]; however, no systematic evaluations have investigated this aspect.

The CSF lactate exam is simple, objective and affordable [6]. The exam is not affected by blood contamination of the CSF [13, 14]. Many researchers also reported that the CSF lactate concentration was not related to the neutrophil count [6, 15, 16]. The test can be performed at bedside, and the results can be received within 15 min. Additionally, a rapid decrease in the CSF lactate level following antibiotic treatment could suggest a relatively good prognosis. Therefore, CSF lactate may play a significant role in the diagnosis of post-neurosurgical bacterial meningitis. This study performed a systematic evaluation and meta-analysis of the efficacy of CSF lactate concentration in diagnosing post-neurosurgical bacterial meningitis.

## Methods

### Review of ethics committee

This study is a meta-analysis based on published data from previous studies. Hence, no review by an ethics committee needed.

### Standard of systematic reviews

This study is designed and performed according to the “Transparent reporting of systematic reviews and meta-analyses” (PRISMA) guidelines.

### Inclusion and exclusion criteria

This analysis included studies that were published in international journals and investigated the CSF lactate concentration in the diagnosis of post-neurosurgical bacterial meningitis.

The inclusion/exclusion criteria were as follows: (1) The study’s objectives included an evaluation of the diagnostic value of the CSF lactate concentration in post-neurosurgical bacterial meningitis. (2) The study subjects were patients who underwent neurosurgery. Studies irrelevant to neurosurgery, studies on patients who did not undergo neurosurgery, and animal studies were excluded from this analysis. (3) The studies used etiological methods as basic diagnostic tools, which means that a Gram stain or bacterial culture of CSF was considered the basis of the gold diagnostic standard for postoperative bacterial meningitis. (4) A diagnostic test fourfold table was included in the studies or could be indirectly obtained by calculations utilizing the data provided in the studies. (5) The studies were published in English.

### Measured parameters

The indexes included sensitivity, specificity, positive likelihood ratio (LR+), negative likelihood ratio (LR-), diagnostic odds ratio (DOR), area under the curve (AUC) of the summary receiver operating characteristics (SROC) curve, and Q* (Q* is the intersection point on the SROC curve where the sensitivity equals specificity).

### Literature retrieval, collection, and screening

We searched PubMed, EBSCO, Cochrane and ClinicalTrials.gov. All the databases were searched from inception to November 2015. (“Lactate” OR “lactic acid”) AND “meningitis” were used as search terms in both PubMed and EBSCO. To avoid ignoring valuable studies, “Lactate” OR “lactic acid” were used to search studies in ClinicalTrials.gov and the Cochrane library.

Studies were selected from the results of the database search. The title and abstract were read first. For each study that could offer a valuable contribution to this analysis and that could not be excluded based on reading only the title and abstract, the full text of the study was directly assessed. If the original text could not be directly accessed, we acquired the full text by contacting the study authors, performing repeated retrievals, searching Google Scholar, or other legal methods.

**Fig. 1:**
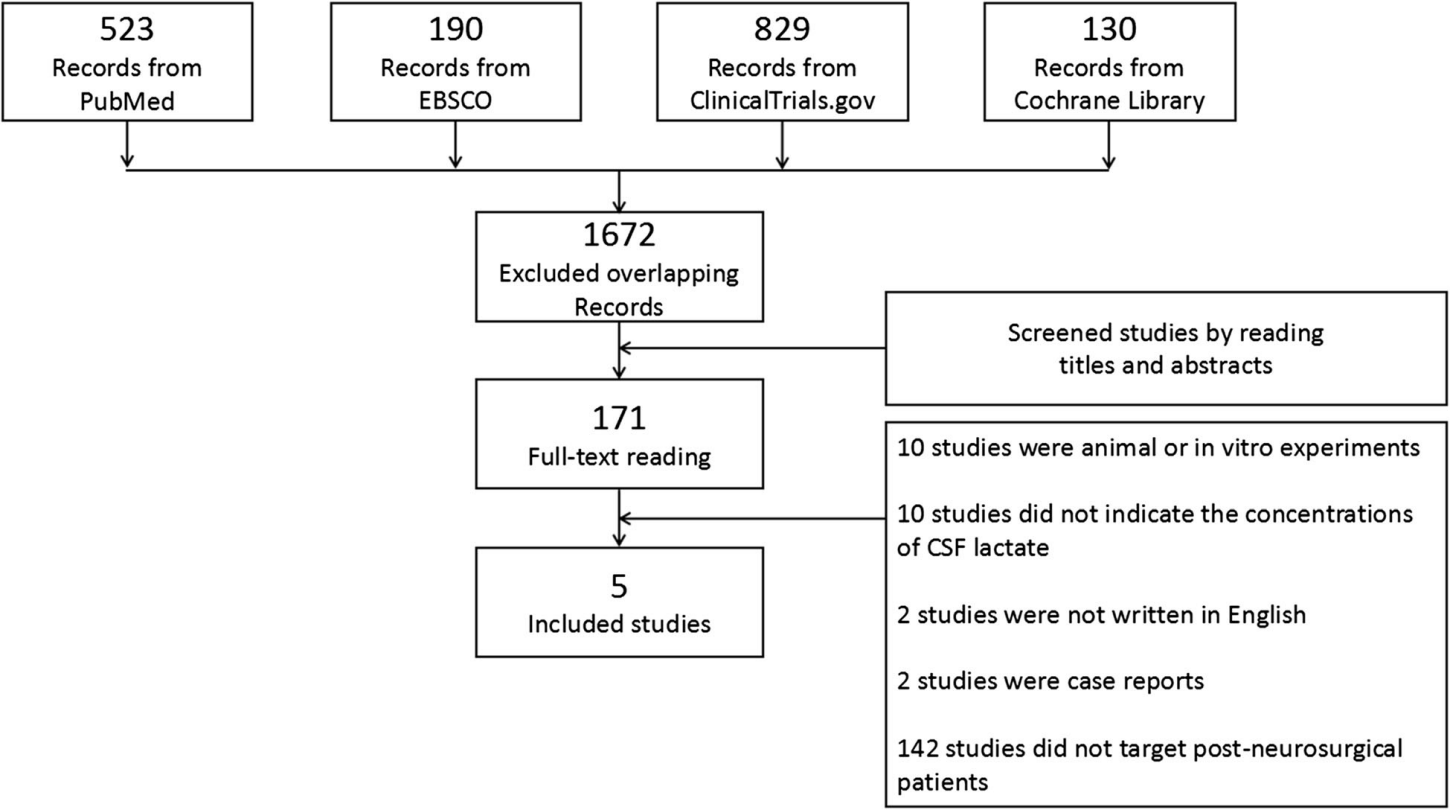
Flow diagram of study screening and selection for inclusion. PRISMA flow diagram of our meta-analysis. A total of 523 studies were obtained from PubMed, 190 studies from EBSCO, 130 studies from the Cochrane library, and 829 from ClinicalTrials.gov. After excluding overlapping literature, 1,672 studies were retrieved. We finally included 5 studies in this analysis according to the above inclusion and exclusion criteria

According to the above study inclusion and exclusion criteria, two investigators (Xiong Xiao and Yang Zhang) performed the literature search and independently acquired and read the studies to exclude articles that were confirmed to hold no useful information for this analysis. The two investigators crosschecked the results of the literature search, discussed the search results and constructed a table for the included studies. A PRISMA flow diagram was drawn (Fig. 1).

### Quality assessment of studies

We evaluated the study quality using the Quality Assessment of Diagnostic Accuracy Studies (QUADAS) method, which contains 14 items for study assessment. Three criteria, “Yes” (satisfies this criterion), “No” (does not satisfy this criterion or was not mentioned) and “unclear” (criterion partially satisfied or cannot obtain sufficient information from the study), were applied to identify the causes of study bias and variations.

### Data extraction

Two investigators independently performed the data extraction, which was consistent. We extracted parameters such as the first author’s name, the publication year, the location of the study, the type of study and the cut-off value of each included study. If the study presented data regarding the number of true positives, number of false positives, number of true negatives, number of false negatives, sensitivity, or specificity, we recalculated these data to ensure correctness. Otherwise, we derived these data according to the related numbers in the included article. All the extracted data were double-checked to avoid errors.

### Statistical analysis

1) We first assessed the heterogeneity of the studies: if the heterogeneity I^2^ > 50 % and *P* < 0.05, heterogeneity was considered to be significant; otherwise, heterogeneity was not significant. When the heterogeneity was significant, a Spearman correlation coefficient was computed between the logit of sensitivity and the logit of (1 − specificity) to assess the threshold effect. 2) A pooled model analysis was chosen according to the results of the heterogeneity assessment. A random effects model was selected if there was significant heterogeneity; otherwise, a fixed effects model was used. 3) The Review Manager 5.2 (Cochrane Editorial Unit, London, UK) and Meta-DiSc 1.4 (Clinical Biostatistics Unit, Ramon y Cajal Hospital, Madrid, Spain) software programs were used for analysis. Forest plots and SROC cures were plotted. The pooled sensitivity, pooled specificity, pooled positive likelihood ratio (LR+), pooled negative likelihood ratio (LR-), pooled diagnostic odds ratio (DOR) and AUC were computed using the software indicated above. Additionally, a Begg’s funnel plot was generated using STATA 12 (StataCorp LP, College Station, TX, USA) to show potential publication bias.

## Results

### Search results

**Fig. 2:**
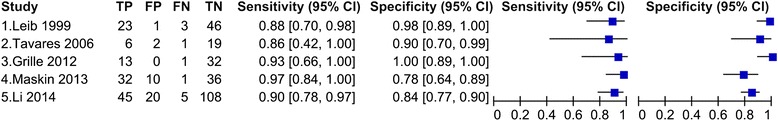
Forest plot drawn using data from the included studies. Forest plots were drawn according to the results of the meta-analysis using Review Manager 5.2 software. The data extracted from the 5 included studies are shown. TP: true positive; FP: false positive; FN: false negative; TN: true negative

After excluding overlapping literature, 1,672 studies were retrieved (Fig. 1), among which 523 studies were obtained from PubMed; 190 studies came from EBSCO; 130 studies were obtained from the Cochrane library; and 829 came from ClinicalTrials.gov. We finally included 5 studies in this analysis according to the above inclusion and exclusion criteria, all of which were published in English and involved a total of 404 post-neurosurgical patients. The publication year of these studies ranged from 1999 to 2014. Among the 5 included studies, 3 were prospective, while the other 2 were retrospective. The cutoff values for CSF lactate ranged from 3.45 mmol/L to 5.4 mmol/L (4.41 ± 0.85 mmol/L). The gold standards of these 5 studies are also shown. One study used only CSF bacterial culture or Gram staining to diagnose post-neurosurgical bacterial meningitis, while the other 4 studies employed diagnostic methods including CSF white blood cells (WBC), the CSF neutrophil percentage, or CSF glucose levels (Table 1). The data from fourfold tables for diagnostic tests were extracted from the studies and prepared for the subsequent analysis (Fig. 2).

**Table 1:** Studies included in this meta-analysis

Authors	Publication year	Type of study	Cut-off value (mmol/L)	Gold standard
Leib et al. [6]	1999	Retrospective	4.0	1) or 2) or 3): 1) positive bacterial CSF culture and CSF WBC > 2.5 × 10^8^/L 2) CSF WBC > 1 × 10^9^/L and neutrophils >50 % 3) CSF WBC > 2.5 × 10^8^/L and neutrophils >50 % in patients treated with steroids or antibiotics at the time of LP
Tavares et al. [25]	2006	Prospective	5.4	positive bacterial CSF culture or Gram stain
Grille et al. [21]	2012	Prospective	5.2	1) or 2): 1) positive bacterial CSF culture or Gram stain 2) negative bacterial CSF culture or Gram stain and CSF WBC > 1 × 10^9^/L (>50 % neutrophils) in patients treated with antibiotics at the time of lumbar puncture
Maskin et al. [22]	2013	Prospective	4.0	1) or 2): 1) positive bacterial CSF culture or Gram stain and CSF WBC ≥ 1 × 10^5^/L or (CSF glucose <40 mg/dL or CSF glucose/blood glucose <0.4) 2) CSF WBC ≥ 2.5 × 10^5^/L and CSF glucose/blood glucose <0.5 if patients received antibiotics 24 h prior to CSF sampling
Li et al. [18]	2014	Retrospective	3.45	^a^ All of the below: 1) clinical symptoms 2) positive bacterial CSF culture or Gram stain 3) CSF WBC count ≥1 × 10^9^/L and polykaryocyte percentage ≥75 % 4) CSF glucose <2.5 mmol/L or CSF glucose/blood glucose <0.4.

^a^ Patients who did not meet these criteria with a CSF WBC count <5 × 10^8^/L were classified into the non-PNBM group

We evaluated the quality of the included studies using the QUADAS tool, the results are presented in Table 2.

**Table 2:** Quality assessment of the included studies according to QUADAS

	1	2	3	4	5	6	7	8	9	10	11	12	13	14
Leib 1999 [6]	Yes	Yes	Unclear	Yes	Yes	Yes	Yes	Yes	Yes	Unclear	Yes	Yes	No	No
Tavares 2006 [25]	Yes	Yes	Unclear	Yes	Yes	Yes	Yes	Yes	Yes	Unclear	Unclear	Yes	No	No
Grille 2012 [21]	Yes	Yes	Unclear	Yes	Yes	Yes	Yes	Yes	Yes	Unclear	Unclear	Yes	No	No
Maskin 2013 [22]	Yes	Yes	Unclear	Yes	Yes	Yes	Yes	Yes	Yes	Unclear	Unclear	Yes	No	No
Li 2014 [18]	Yes	Yes	Unclear	Yes	Yes	Yes	Yes	Yes	Yes	Unclear	Unclear	Yes	No	No

### Analysis results

**Fig. 3:**
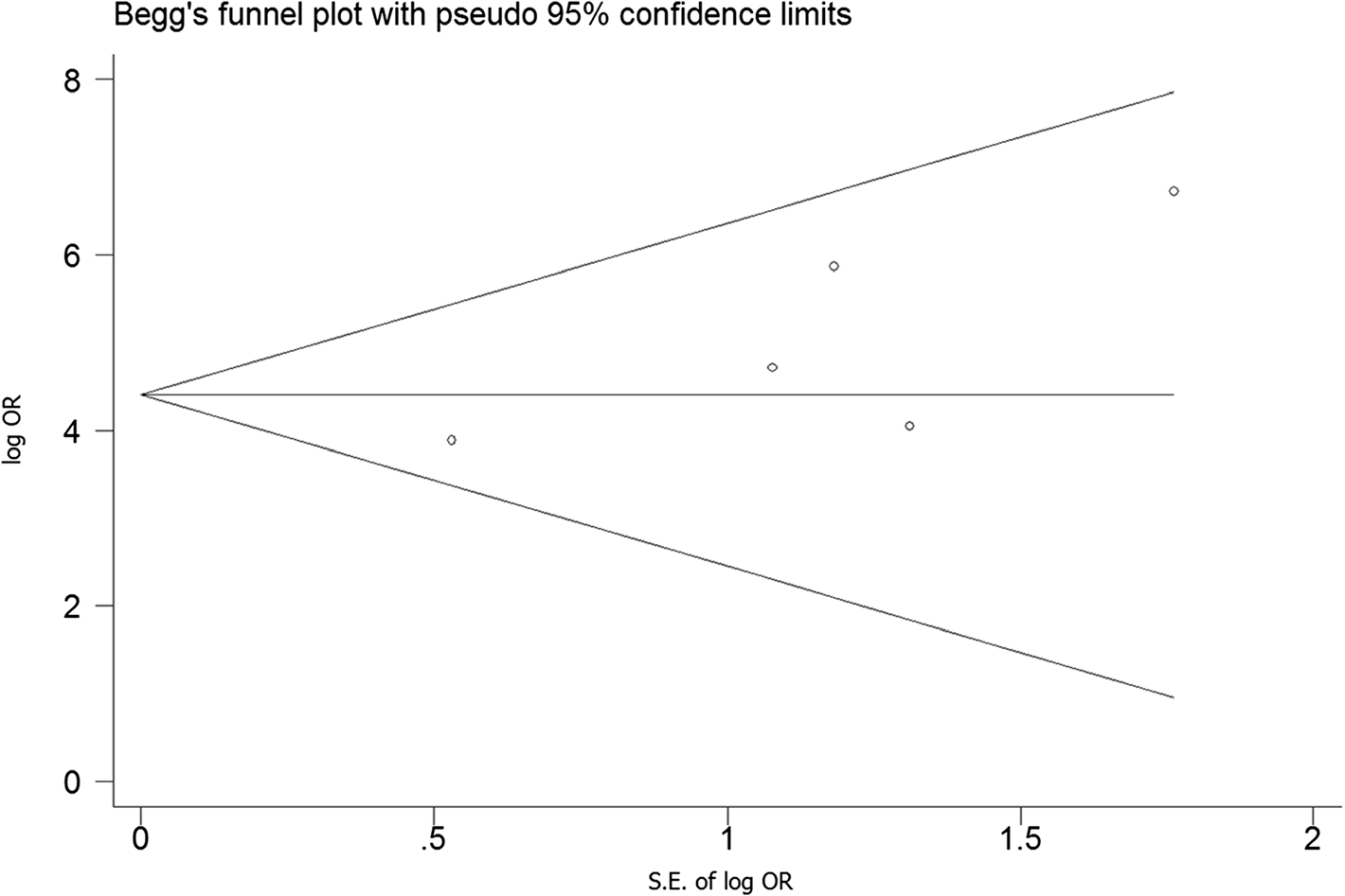
Begg’s funnel plot. Begg’s funnel plot was drawn using STATA 12. Each circle means that a study included. OR: odd ratio; S.E.: standard error

Forest plots were drawn according to the results of the meta-analysis using the Review Manager 5.2 software (Fig. 2). A Begg’s funnel plot was drawn using STATA 12 (Fig. 3) to assess publication bias. All of the dots were within the 95 % CI with a symmetrical distribution in an approximate funnel shape.

**Fig. 4:**
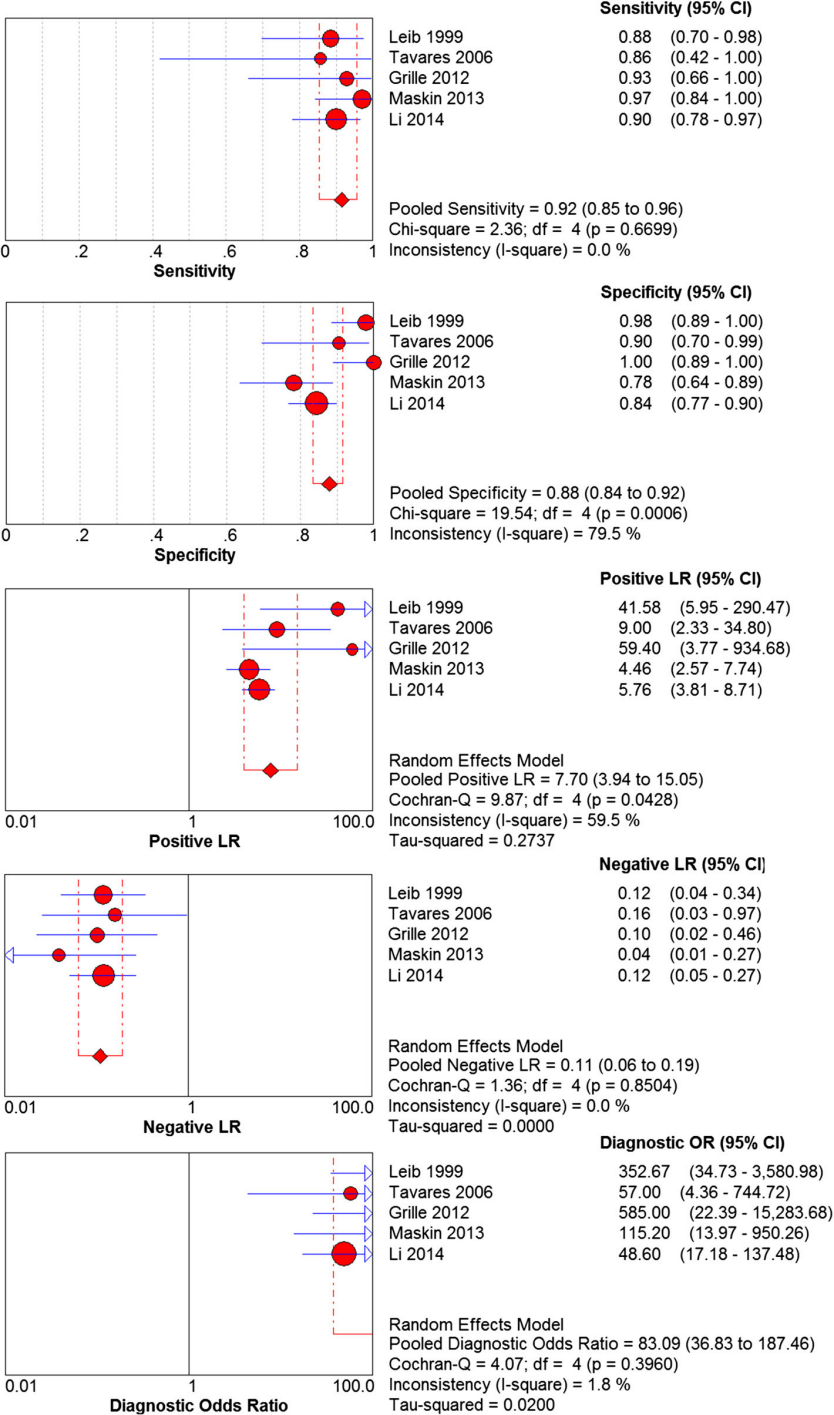
Results from the pooled analysis of the included studies. Results of the pooled analysis using a random effects model. The sensitivity, specificity, positive LR, negative LR, and diagnostic OR of all of the included studies were pooled. The pooled sensitivity was 0.92 (95 % CI 0.85–0.96); the pooled specificity was 0.88 (95 % CI 0.84–0.92, with significant heterogeneity); and the diagnostic OR was 83.09 (95 % CI 36.83–187.46). LR: likelihood ratio

**Fig. 5:**
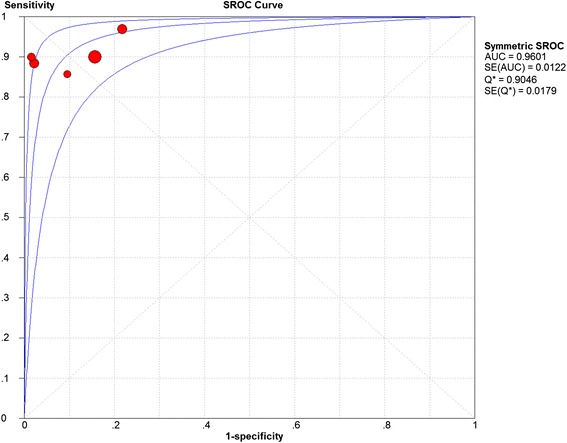
SROC Curve. SROC curves were plotted. Each red point represent an included study. The AUC of the SROC curve was 0.9601, and the Q* was 0.9046. SROC: summary receiver operating characteristic; AUC: area under curve; Q*: the point on the SROC where sensitivity equals specificity

It can be seen from the analysis that the heterogeneity of the 5 studies (I2) was 79.5 %, (*P* = 0.0006). This heterogeneity was significant. The Spearman correlation coefficient between the logit of sensitivity and the logit of (1 − specificity) was 0.462 (*P* = 0.434), indicating that there was no obvious threshold effect in the estimates of accuracy. Therefore, the random effects model was chosen for the pooled analysis. Meta-DiSc 1.4 was used to select the random effects model for the pooled analysis. The results were as follows: the pooled sensitivity was 0.92 (95 % CI 0.85–0.96), the pooled specificity was 0.88 (95 % CI 0.84–0.92 with significant heterogeneity), the pooled LR+ was 7.70 (95 % CI 3.94–15.05 with significant heterogeneity), the pooled LR- was 0.11 (95 % CI 0.06–0.19), the pooled DOR was 83.09 (95 % CI 36.83–187.46), the AUC of the SROC curve was 0.9601 and the Q* was 0.9046 (Figs. 4 and 5).

## Discussion

When performing this systematic review and meta-analysis, we found only a few diagnostic studies on post-neurosurgical intracranial infection, and two of these were retrospective studies. Few high-quality clinical trials or prospective studies have been conducted. The diagnosis and treatment of intracranial infection has become a critical and urgent problem in neurosurgery. Thus, it is necessary to conduct related clinical research to develop highly specific, sensitive, convenient, fast and affordable early diagnostic indexes. Additionally, scientific and systematic evaluations of the diagnostic efficacy of these indexes should be performed. This work has great significance for the early diagnosis and treatment of post-neurosurgical bacterial meningitis.

According to our results, the CSF lactate exam shows relatively good efficacy. Moreover, this test is fast, simple, objective, and affordable and can be widely applied in hospitals.

Several factors may affect the results of this meta-analysis: 1) A small number of studies, only 5, were included. Moreover, two of these were retrospective studies. The number of study subjects was relatively small (only 404 patients). 2) It was difficult to standardize the CSF lactate measurement exam methods among the 5 studies. 3) Two of the included studies defined the cutoff value before analysis; however, the other three generated the value based on the highest sensitivity and specificity of the ROC curve. The cutoff values in the studies ranged from 3.45 mmol/L to 5.4 mmol/L. Although the Spearman correlation between the logit of sensitivity and the logit of (1 − specificity) showed that there was no obvious threshold effect, the situation would be improved if more studies using same cutoff value were available. 4) Of the 5 included studies, only 1 used etiological examination as the gold standard for diagnosis; the other 4 studies included patients with clinically confirmed diagnoses, which means that the patients had a negative etiology test result but presented with significant clinical symptoms and signs and that their cerebrospinal cell analysis exhibited significantly abnormal results.

Additionally, we found that the CSF lactate cutoff value was defined before the analysis in 2 studies; however, in the others, it was defined based on the best value according to the ROC curve. To determine whether this difference was responsible for the heterogeneity of the studies, we used Meta-DiSc 1.4 to run a Meta-Regression. The results indicated that this difference did not cause the observed heterogeneity (*P* = 0.3141). To further analyze the sources of heterogeneity, we ran a subgroup analysis based on the type, publication year, sample size, and diagnostic method of the studies and whether the cutoff values were defined before analysis. It was found that dividing the studies into two groups based on whether the publication year was before 2013 reduced the heterogeneity in each subgroup. The pooled diagnostic values in the subgroup with earlier publication dates were as follows: sensitivity, 0.894 (95 % CI: 0.769–0.965); specificity, 0.970 (95 % CI: 0.915–0.994); LR+, 21.442 (95 % CI: 5.330–86.101); LR-, 0.120 (95 % CI: 0.054–0.263); DOR, 207.71 (95 % CI: 45.321–951.99); AUCSROC, 0.9795; Q*, 0.9363. The pooled diagnostic values in the subgroups with later publication dates were as follows: sensitivity, 0.928 (95 % CI: 0.849–0.973); specificity, 0.828 (95 % CI: 0.763–0.881); LR+, 5.255 (95 % CI: 3.775–7.313); LR-, 0.095 (95 % CI: 0.040–0.231); DOR, 57.528 (95 % CI: 22.636–146.21); AUCSROC, 0.9469; Q*, 0.8864.

Although positive CSF culture or Gram stain results are the most direct evidence of infection, the percentages of the positive results are low for a variety of reasons, including the low volume, contamination of the CSF sample, time constraints and antibiotic drug administration [17, 18]. This makes the “gold standard” less sensitive and specific, leading to low diagnostic efficacy. It is possible to misdiagnose many patients with postoperative infections when using only the etiological exam to confirm diagnosis. This would result in higher mortality and study bias. Additionally, the CSF culture requires several days to generate a result, which is sometimes too long to wait for treatment. Etiological examinations in addition to CSF cytology and biochemical examinations are widely used to achieve better efficacy. However, there is no specific criterion for the threshold values of the involved items. In other words, there is no other gold standard. As Li reported, the widely used criteria for diagnosing post-neurosurgery bacterial meningitis include those proposed by the Centers for Disease Control and Prevention [19], the Massachusetts General Hospital [20], the Infectious Diseases Society of America and the Beijing Tiantan Hospital [18]. Four of the 5 studies that we included also used standards that differed from those of other studies. However, the evidence indicating whether the patients were suffering from post-neurosurgical meningitis was objective. Regardless of the criterion, meeting the established standard meant a greater chance that the post-neurosurgical meningitis diagnosis was correct. Moreover, the studies we included compared the CSF lactate levels of patients whose diagnoses were etiologically confirmed with those whose diagnoses were clinically confirmed (i.e., those with negative etiological diagnoses but with the presence of significant clinical manifestations and abnormal CSF levels); there was no statistically significant difference between these patients [6, 21, 22].

New techniques, such as polymerase chain reaction (PCR), can improve diagnostic sensitivity; however, these techniques are usually unavailable to most hospitals or laboratories. New diagnostic markers, such as PCT, sCD163 and CRP, have also been reported [18, 23, 24]. Furthermore, some researchers tested the efficacy of combinations of markers and obtained optimistic results [18]. However, most studies concerning these markers are retrospective or have small sample sizes.

Neurosurgery involves the management of many types of diseases. It may not be appropriate to treat these diseases equally and to put all of the post-operative conditions into a single category (“post-neurosurgical”) when investigating post-operative infections. The operative procedures vary according to differences in disease type, lesion location, patient condition and other factors. In the design period, we attempted to search for articles on “post-craniotomy meningitis” because we believed that the infections in patients who received craniotomies would differ from those in patients who did not undergo craniotomy. However, the article retrieval results were not optimistic.

This meta-analysis summarized the studies targeting the diagnostic efficacy of CFS lactate in post-neurosurgical bacterial meningitis and reached a relevant conclusion. However, more high-quality, large-scale, prospective studies are required to obtain a more thorough understanding of and better biomarkers or indicators for post-neurosurgical meningitis.

## Conclusion

This meta-analysis investigated the diagnostic value of CSF lactate for post-neurosurgical bacterial meningitis. We included 5 studies on a total of 404 post-neurosurgical patients to obtain pooled data. The results indicate that the CSF lactate concentration shows a relatively high sensitivity and specificity in the diagnosis of post-neurosurgical bacterial meningitis and thus has relatively good efficacy. However, additional precise, accurate and convincing data from prospective studies and from those with large samples is necessary.

## Abbreviations

CSF: Cerebrospinal fluid
QUADAS: Quality assessment of diagnostic accuracy studies
SROC: Summary receiver operating characteristics
LR+: Positive likelihood ratio
LR-: Negative likelihood ratio
OR: Odds ratio
S.E.: Standard error
WBC: White blood cells

## References

[1] Blomstedt GC (1985). Infections in neurosurgery: a retrospective study of 1143 patients and 1517 operations. Acta Neurochir.

[2] Ross D, Rosegay H, Pons V (1988). Differentiation of aseptic and bacterial meningitis in postoperative neurosurgical patients. J Neurosurg.

[3] Kaufman BA, Tunkel AR, Pryor JC, Dacey RG (1990). Meningitis in the neurosurgical patient. Infect Dis Clin N Am.

[4] Carmel PW, Greif LK (1993). The aseptic meningitis syndrome: a complication of posterior fossa surgery. Pediatr Neurosurg.

[5] Wang KW, Chang WN, Huang CR, Tsai NW, Tsui HW, Wang HC (2005). Post-neurosurgical nosocomial bacterial meningitis in adults: microbiology, clinical features, and outcomes. J clin neurosci.

[6] Leib SL, Boscacci R, Gratzl O, Zimmerli W (1999). Predictive value of cerebrospinal fluid (CSF) lactate level versus CSF/blood glucose ratio for the diagnosis of bacterial meningitis following neurosurgery. Clin infect dis.

[7] Kumar A, Roberts D, Wood KE, Light B, Parrillo JE, Sharma S (2006). Duration of hypotension before initiation of effective antimicrobial therapy is the critical determinant of survival in human septic shock. Crit Care Med.

[8] Genton B, Berger JP (1990). Cerebrospinal fluid lactate in 78 cases of adult meningitis. Intensive Care Med.

[9] Pavese P, Francois P, Lafond JL, Kayemba Kay SS, Bosson JL (1997). Assay of lactic acid in the cerebrospinal fluid for the diagnosis of bacterial meningitis. Strategies for the choice of discriminatory threshold. Presse Med.

[10] Huy NT, Thao NT, Diep DT, Kikuchi M, Zamora J, Hirayama K (2010). Cerebrospinal fluid lactate concentration to distinguish bacterial from aseptic meningitis: a systemic review and meta-analysis. Crit Care.

[11] Sakushima K, Hayashino Y, Kawaguchi T, Jackson JL, Fukuhara S (2011). Diagnostic accuracy of cerebrospinal fluid lactate for differentiating bacterial meningitis from aseptic meningitis: a meta-analysis. J infect.

[12] van de Beek D, Drake JM, Tunkel AR (2010). Nosocomial bacterial meningitis. N Engl J Med.

[13] Begovac J, Bace A, Soldo I, Lehpamer B (1991). Lactate and glucose in cerebrospinal fluid heavily contaminated with blood. Acta medica Croatica.

[14] Cameron PD, Boyce JM, Ansari BM (1993). Cerebrospinal fluid lactate in meningitis and meningococcaemia. J infect.

[15] Bland RD, Lister RC, Ries JP (1974). Cerebrospinal fluid lactic acid level and pH in meningitis. Aids in differential diagnosis. Am J Dis Child.

[16] Jordan GW, Statland B, Halsted C (1983). CSF lactate in diseases of the CNS. Arch Intern Med.

[17] Simon TD, Pope CE, Browd SR, Ojemann JG, Riva-Cambrin J, Mayer-Hamblett N (2014). Evaluation of microbial bacterial and fungal diversity in cerebrospinal fluid shunt infection. PLoS One.

[18] Li Y, Zhang G, Ma R, Du Y, Zhang L, Li F (2015). The diagnostic value of cerebrospinal fluids procalcitonin and lactate for the differential diagnosis of post-neurosurgical bacterial meningitis and aseptic meningitis. Clin Biochem.

[19] Korinek AM, Baugnon T, Golmard JL, van Effenterre R, Coriat P, Puybasset L (2006). Risk factors for adult nosocomial meningitis after craniotomy: role of antibiotic prophylaxis. Neurosurgery.

[20] Durand ML, Calderwood SB, Weber DJ, Miller SI, Southwick FS, Caviness VS (1993). Acute bacterial meningitis in adults. A review of 493 episodes. N Engl J Med.

[21] Grille P, Torres J, Porcires F, Bagnulo H (2012). Value of cerebrospinal fluid lactate for the diagnosis of bacterial meningitis in postoperative neurosurgical patients. Neurocirugia.

[22] Maskin LP, Capparelli F, Mora A, Hlavnicka A, Orellana N, Diaz MF (2013). Cerebrospinal fluid lactate in post-neurosurgical bacterial meningitis diagnosis. Clin Neurol Neurosurg.

[23] Knudsen TB, Larsen K, Kristiansen TB, Moller HJ, Tvede M, Eugen-Olsen J (2007). Diagnostic value of soluble CD163 serum levels in patients suspected of meningitis: comparison with CRP and procalcitonin. Scand J Infect Dis.

[24] Shimetani N, Shimetani K, Mori M (2001). Levels of three inflammation markers, C-reactive protein, serum amyloid A protein and procalcitonin, in the serum and cerebrospinal fluid of patients with meningitis. Scand J Clin Lab Invest.

[25] Tavares WM, Machado AG, Matushita H, Plese JP (2006). CSF markers for diagnosis of bacterial meningitis in neurosurgical postoperative patients. Arq Neuropsiquiatr.

